# Harnessing Real-World Evidence to Advance Cancer Research

**DOI:** 10.3390/curroncol30020143

**Published:** 2023-02-02

**Authors:** Monica Tang, Sallie-Anne Pearson, Robert J. Simes, Boon H. Chua

**Affiliations:** 1 Nelune Comprehensive Cancer Centre, Prince of Wales Hospital, Randwick 2031, Australia; 2 School of Population Health, UNSW Sydney, Sydney 2052, Australia; 3 NHMRC Clinical Trials Centre, University of Sydney, Camperdown 2050, Australia; 4 Faculty of Medicine and Health, UNSW Sydney, Sydney 2052, Australia

**Keywords:** real-world evidence, observational study, randomized controlled trials, oncology

## Abstract

Randomized controlled trials (RCTs) form a cornerstone of oncology research by generating evidence about the efficacy of therapies in selected patient populations. However, their implementation is often resource- and cost-intensive, and their generalisability to patients treated in routine practice may be limited. Real-world evidence leverages data collected about patients receiving clinical care in routine practice outside of clinical trial settings and provides opportunities to identify and address gaps in clinical trial evidence. This review outlines the strengths and limitations of real-world and RCT evidence and proposes a framework for the complementary use of the two bodies of evidence to advance cancer research. There are challenges to the implementation of real-world research in oncology, including heterogeneity of data sources, timely access to high-quality data, and concerns about the quality of methods leveraging real-world data, particularly causal inference. Improved understanding of the strengths and limitations of real-world data and ongoing efforts to optimise the conduct of real-world evidence research will improve its reliability, understanding and acceptance, and enable the full potential of real-world evidence to be realised in oncology practice.

## 1. Introduction

The growing incidence and burden of cancer drives the need for effective, evidence-based treatments. Globally, there were 18.1 million cancer cases and 9.6 million deaths due to cancer in 2018, with the annual number of cases projected to increase to 29 million by 2040 [1]. As the global burden of cancer increases, so does the economic cost of cancer treatment. In the United States, cancer healthcare spending was estimated to be USD 161.2 billion in 2017 [1], compared to USD 27 billion in 1990 [2]. In Europe, the total cost of cancer care was EUR 199 billion in 2018, comprising EUR 103 billion on healthcare spending, EUR 26 billion on informal care costs, and EUR 70 billion on productivity losses [3].

**Figure 1:**
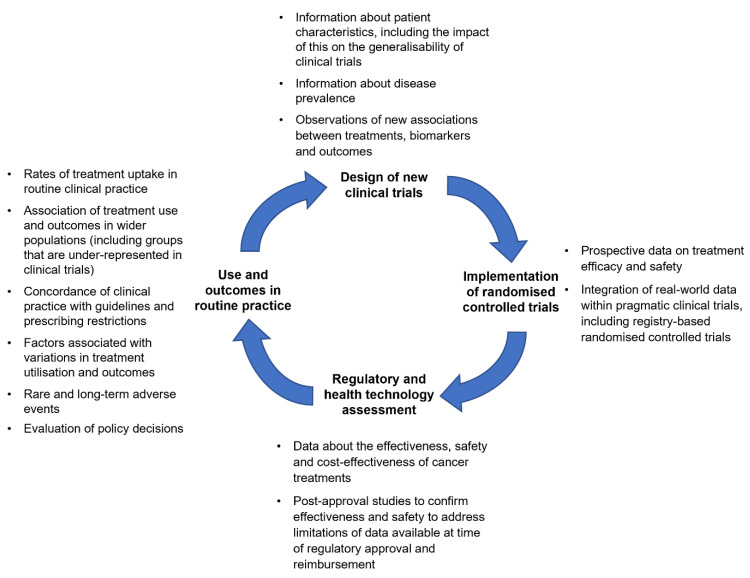
Life cycle of cancer medicine development and areas where evidence is needed.

In this context, reliable evidence is required to support the development and use of cancer medicines. Figure 1 is a schema of the iterative process of cancer medicine development, and areas where evidence is needed to drive high-quality care and optimize outcomes for people with cancer. While clinical trials are integral to the development of novel cancer therapies, there is increasing recognition that conventional trials may not meet all of the evidentiary needs for regulatory assessment and clinical decision-making. This has led to growing interest in the potential of real-world data to generate fit-for-purpose real-world evidence in cancer care.

This review outlines the strengths and limitations of conventional clinical trials and real-world data for oncology research and explores the potential roles of real-world data for addressing evidence gaps in clinical trial research. This is followed by a proposed framework for the complementary use of clinical trials and real-world evidence to advance oncology research, and a targeted overview of the potential of real-world data globally to support cancer research.

## 2. Conventional Clinical Trials and Real-World Data Studies

Conventional clinical trials and studies leveraging real-world data represent two broad categories of evidence generation for cancer therapies, each with their respective strengths and limitations (Table 1).

### 2.1. Clinical Trials

Randomized controlled trials (RCTs) evaluate the efficacy and toxicity of novel treatments against standard-of-care comparators and are considered a “gold standard” for determining whether there is a cause-effect relationship between treatment and outcome [4]. RCTs are usually Phase 3 trials that aim to demonstrate a statistically significant difference between two or more treatment arms, with the alpha error conventionally set at 0.05, indicating a 5% risk of rejecting the null hypothesis when it is true. Well-designed RCTs enable an even distribution of factors, both known and unknown, that may affect the outcome between treatment groups, to minimize the effect of confounding bias on outcomes of interest. To further reduce bias, RCTs employ strategies such as allocation concealment, blinded assessment, intention-to-treat analysis and rigorous follow-up, so that differences in outcomes between treatment groups can be attributed to the intervention under investigation [4,5]. Therefore, the major strength of RCTs is the ability to evaluate the efficacy of interventions with excellent internal validity (Table 1).

However, RCTs have limited external validity, as the generalizability of their outcomes is constrained by several factors, including differences in patient population and care provision between the clinical trial and real-world settings [6,7].

1. Clinical trial participants are highly selected and may not be representative of the broader patient population, as only 3% of patients with cancer are enrolled in RCTs [8,9]. Older patients aged 65 years and above are consistently under-represented in clinical trials, even though cancers are overwhelmingly diagnosed in older adults [9,10,11,12,13,14]. There is also under-representation of patients from racial and ethnic minorities, those with socioeconomic disadvantage, and patients with complex health problems; therefore, RCTs may lack information about treatment tolerability and efficacy in patients with multiple co-morbidities and poor performance status [9,15,16,17,18].
2. Patient care provided in clinical trials does not necessarily represent routine clinical practice [6,19]. Clinical trial participants typically receive more intensive monitoring than patients in routine practice, which may influence outcomes. There is evidence that clinical trial participants benefit from the ‘trial effect’ or ‘protocol/Hawthorne effect’, in which the clinical trial participation in itself may have a positive effect on outcomes due to more intensive care [20,21]. This is supported by the fact that patients who are referred for clinical trial participation at specialist centres often have better survival outcomes than those who are not [22,23].

RCTs have a number of practical limitations, including the need to prospectively recruit and monitor study participants, often in large numbers in highly controlled processes that are costly, cumbersome and time-consuming. A report using pharmaceutical industry data from over 4100 oncology trials found that the average duration of phase III oncology trials was nearly 5 years [24]. Data on key clinical outcomes, such as overall survival, can take many years to mature. This often leads to the use of surrogate endpoints that can be available in shorter time frames, such as response rate and progression-free survival. Between 2009 and 2014, approval for two-thirds of oncology drugs by the United States Food and Drug Administration (FDA) were based on surrogate outcomes [25,26]. However, many RCTs employ surrogate endpoints that are not adequately validated measures of patient benefit [26]. Furthermore, RCTs are limited in terms of patient numbers and duration of follow-up. Therefore, RCTs have a limited ability to provide data on rare and long-term toxicities, especially in patients who are under-represented in clinical trials or those that occur many years after study completion [19].

### 2.2. Real-World Data

Real-world data are not generated by conventional RCTs [27]. A widely accepted definition of real-world data is proposed by the United States Food and Drug Administration (FDA) in the Framework for FDA’s Real-World Evidence Program: “data relating to patient health status and/or delivery of health care routinely collected from a variety of sources” [28]. Real-world evidence describes information on healthcare derived from real-world data settings. Its defining characteristics are the routine care settings in which data are collected and the degree of pragmatism [29,30].

#### 2.2.1. Examples of Real-World Data

Real-world data is heterogeneous (Table 2). Electronic health records (EHR) in routine care have created a rich potential source of real-world data. Health claims datasets consist of data on billing and payment interactions between patients and health care providers and payers, collected for the purposes of reimbursement. Health surveys are conducted to provide information on the health of populations and are administered at regular intervals on a random sample of individuals or households [27,31]. Registries are population-specific, prospective, observational collections of predefined clinical, demographic and disease characteristics of patient cohorts who have a particular disease and/or receive a particular treatment or intervention [27]. Cancer registries are a type of disease-based registry recording all new cases of cancer in a defined population [32]. Novel sources of real-world data include patient-generated data, facilitated by the emergence of technologies such as wearable devices, health-related mobile applications and social media platforms [33].

#### 2.2.2. Strengths of Real-World Data Research

An important strength of real-world data is that data are captured from patients in routine care (Table 1). Therefore, studies leveraging these data cover a broader cross-section of the patient population than clinical trials, potentially producing more generalizable results with greater external validity. When relevant data and infrastructure are available, real-world studies can be conducted more efficiently and at a lower cost than conventional clinical trials. Studies using real-world data typically have larger sample sizes and longer periods of follow-up compared to clinical trials, facilitating detection of late and uncommon side-effects. Therefore, real-world data are able to offer insights into larger, more heterogenous patient populations in routine practice, which contrasts with and complements the evidence arising from the study of strictly defined, homogeneous participants in conventional clinical trials.

#### 2.2.3. Limitations of Real-World Data Research

A key limitation of observational studies of treatment efficacy and toxicity is that the intervention of interest is not randomly assigned, and there is often not a suitable control group. Thus, results are susceptible to confounding by indication, which could result in biased associations between treatment and outcomes of interest. Due to the imperfect internal validity of observational studies, it is often difficult to ascertain whether outcomes are due to the adoption of a new treatment, or whether they are due to underlying patient characteristics that influence treatment choice (selection bias), or other factors such as changes in disease biology or concurrent changes in patient management.

Another important limitation of studies using real-world data is that the primary aim for many data collections is to support health service provision or administrative purposes, rather than research purposes. Therefore, real-world data may lack information on research end points, and have more variable data quality and incomplete data compared to conventional clinical trials.

## 3. Potential Uses of Real-World Data in Oncology Research

The variety of real-world data sources is paralleled by the wide range of their potential uses in cancer research [34].

- *Studying patients that are under-represented in clinical trials:* For example, in oncology, the under-representation of older adults in clinical trials leads a relative shortage of evidence to guide their care [9,10,11,12,13,14,35]. Real-world data offer opportunities to study the extent of and factors contributing to evidence gaps for these patients, and to gain insights into their management and outcomes.
- *Examining cancer therapy use and outcomes:* While RCTs offer evidence of what is achievable under favourable circumstances, they do not necessarily provide a reliable indication of outcomes of patients who receive the same interventions in less controlled circumstances [19,34]. Real-world data offers opportunities to examine how routine care differs from clinical trials and trial evidence-based guidelines. Differences in patients, practice and providers often leads to patients in routine practice having shorter survival and higher rates of treatment toxicity compared to clinical trial participants [36,37]. This difference between outcomes of patients selected to participate in trials (efficacy) and outcomes when the same treatment is applied in real-world practice (effectiveness) is referred to as the efficacy-effectiveness gap [38,39].
- *Rare cancers:* It is challenging to generate evidence to guide the care of patients with rare cancers due to difficulty in accruing sufficient participants to RCTs to have adequate statistical power to detect differences in outcomes. Observational studies using real-world data are increasingly recognized as a means to advance research into rare cancers by improving the understanding of their natural history, evaluating clinical practice, establishing standards of care, and generating hypotheses for testing in clinical trials [40,41].
- *Rare and long-term toxicities:* While RCTs have limited ability to provide information on rare and late treatment toxicities, real-world data research often include data from larger numbers of patients collected over longer periods of time and hence could provide this information.
- *Health economic evaluation:* Health economic evaluation is used to model anticipated costs associated with adoption of new cancer medicines and is often used by health technology assessment bodies to determine funding of and access to treatments. However, these predictive models and estimates are often based on assumptions that may not accurately reflect the true costs of health interventions in the real world. Real-world data can enable estimation of actual health care use and costs to support health economic evaluation.

## 4. A Framework for Clinical Trials and Real-World Evidence in Oncology Research

RCTs provide precise estimates of treatment efficacy in select patient populations and controlled settings, while real-world evidence examines effectiveness in broader patient populations and settings but are susceptible to biases in establishing associations and/or causality between treatments and outcomes. Hence, these two bodies of evidence are complementary and should be employed in a framework that leverages their respective strengths [6,19].

### 4.1. Complementary Roles for Clinical Trials and Real-World Evidence

There is increasing interest in using real-world data in comparative effectiveness research to compare treatments in non-randomized studies [42]. Although methods have been developed to minimize the effect of confounders, such as propensity score analysis, multivariable regression analysis and instrumental variable analysis, they cannot completely eliminate bias as they are unable to account for unmeasured confounders [43,44,45]. Thus, RCTs will continue to have a central role in establishing the fundamental efficacy of novel therapies in controlled conditions [46,47]. Real-world evidence should be used to complement and augment clinical trials. This can be achieved in several ways: by extending clinical trial evidence, informing trial design and directly integrating with clinical trials.

#### 4.1.1. Using Real-World Evidence to Extend Clinical Trial Evidence

Once the efficacy of novel cancer therapies has been established in RCTs, real-world studies can extend RCT evidence by evaluating patterns of care and safety profile of the therapy outcomes in typical patients [6,48].

Real-world data can also be leveraged to generate evidence on outcomes that are not typically available from RCTs, such as rare, long-term or unexpected toxicities. Forty percent of potentially fatal adverse drug reactions reported in the post-market setting were not reported in pivotal RCTs, and 60% were not described in initial drug labels [49]. The Sentinel System was launched by the United States FDA in 2008 and integrates routine billing and claims data, electronic health records and registry data from over 200 million patients nationwide [50]. This post-marking surveillance will facilitate the use of real-world evidence to detect new safety signals and extend existing RCT evidence about the safety of cancer medicines [49].

#### 4.1.2. Using Real-World Evidence to Support Clinical Trial Design

Observational studies using real-world data may inform clinical trial design by providing information on characteristics of patients in the general population, or by identifying areas of clinical uncertainty or generating hypotheses that require further investigation in RCTs (Figure 1) [19]. Real-world data can also aid trial design and planning by assisting in the study site selection, providing bases for power calculations, providing a prior for Bayesian statistical analysis, and guiding enrichment [30]. Some of the challenges of Bayesian statistical analysis in clinical trials are that the analyses are usually more complex and also require the selection of a prior probability. However, such a prior can be supported using real-world data.

The burgeoning field of precision oncology aims to develop targeted therapies that are tailored to the molecular profile of each individual’s cancer [51]. Clinico-genomic databases integrate clinical information on patient and treatment characteristics and outcomes with results of genomic analysis to inform precision medicine research, for example, by identifying targets for drug development [52]. Examples of accumulating real-world clinical databases with accompanying genomic data include the American Association for Cancer Research (AACR) Project Genomics Evidence Neoplasia Exchange (GENIE) [53], and the Center for Cancer Genomics and Advanced Therapeutics (C-CAT), the National Datacentre for Cancer Genomic Medicine in Japan [54]. Cancer Learning Intelligence Network for Quality (CancerLinQ) is an example of a real-world data collection, aggregating experiences of off-label use of drugs for indications that are not currently FDA-approved, which may facilitate hypothesis generation and inform the design of future clinical trials [55]. Nevertheless, RCTs are still needed to verify hypotheses based on clinico-genomic observations, as patients are at risk of harm if they receive ineffective or toxic targeted therapies based simply on the presence of detectable molecular targets without robust RCT evidence [56].

#### 4.1.3. Integration of Real-World Evidence in Clinical Trials

Real-world evidence can be integrated in clinical trials to take advantage of the strengths of both observational studies and RCTs. For instance, long-term follow-up of health outcomes can be facilitated by linking trial participants to real-world data sources such as EHR, health administrative claims or registries. In cardiovascular research, studies have demonstrated that linked health administrative claims data have strong agreement with traditional adjudication-based clinical trial endpoints [57,58]. While similar validation studies are yet to be undertaken in oncology, these results suggest that real-world data may provide a feasible method for assessing a treatment effect in oncology RCTs, especially for endpoints such as death, that are reliably captured in health administrative and registry datasets [59].

Pragmatic trials also offer opportunities for incorporating real-world evidence into clinical trials, by providing a link between “efficacy studies” of RCTs and “effectiveness studies” that are relevant to clinical practice [39]. Pragmatic trials randomize treatment allocation but otherwise promote treatment delivery to a broader range of patients in routine practice by staff with typical experience [60,61]. Data collection is achieved through processes that would be in place irrespective of the trial, such as EHRs and disease registries [61]. This enables the study of interventions in a representative population of patients in their usual clinical environment, while providing the statistical benefits of randomization [62].

In registry-based RCTs, patients are identified, recruited and may be followed up via clinical registries [63]. VALIDATE-SWEDEHEART is an example of a cardiovascular registry-based RCT that compared two anticoagulation therapies in patients with myocardial infarction, conducted via Sweden’s national online cardiac registry [64]. In contrast, most registry-based RCTs in oncology to date have focused on preventative interventions, including cancer screening [65]. Registry-based RCTs have been heralded as the “next disruptive technology in clinical research” due to their potential to leverage clinical information that is already gathered in pre-existing registries to facilitate timely identification and enrolment of patients and obtain accurate follow-up information with minimal efforts and costs [66].

### 4.2. Real-World Evidence to Support Population-Level Decision-Making

Real-world evidence is valuable for supporting population access to safe and effective cancer therapies. RCT evidence forms the cornerstone of regulatory and reimbursement decisions, but uncertainties remain regarding the reliability of their results. Walsh et al. proposed the Fragility Index as a measure of the statistical robustness of clinical trial results, by estimating the number of additional events that are required to turn a statistically significant result to non-significant [67]. Many RCTs supporting FDA-approved anti-cancer medicines have a low Fragility Index, which means that the statistical significance of their results could be reversed with a small number of additional events [68]. This highlights the uncertainties around the robustness of RCT results, which should be addressed using post-marketing studies to ensure that statistically significant efficacy reported in RCTs translates to effectiveness in clinical practice (Figure 1) [42,69].

Medicine approval and reimbursement decisions are increasingly based on single-arm, non-randomised studies reporting preliminary data or surrogate endpoints, often with the expectation of post-approval data collection to corroborate results [70]. Between 1992 and 2017, the FDA granted accelerated approval to 64 malignant hematology and oncology products for 93 new indications, of which single-arm trial designs provided the data for 72% of the initial indications [71]. Among United States patients receiving FDA-approved novel oral targeted cancer medicines, the proportion of patients receiving drugs without a documented overall survival benefit increased from 12.7% in 2011 to 58.8% in 2018 [72]. As the use of accelerated approval programs grows and evidence standards for approval and reimbursement shift towards accepting earlier stage clinical trial data and surrogate outcomes, it becomes more crucial to conduct timely post-approval studies using real-world evidence to confirm the clinical benefit of cancer medicines, and address limitations of the data available at the time of market entry [73,74].

Driven by the need for evidence of effectiveness, cost-effectiveness and safety in real-world settings, and recognition of the limitations of clinical trial evidence, regulatory bodies and health technology assessment agencies increasingly use real-world evidence to address questions that have previously been examined in RCTs [75]. In the United States, enactment of the 21st Century Cures Act in 2016 tasked the FDA with establishing a program to evaluate the potential use of real-world evidence to support the approval of new indications for approved medicines or to satisfy post-approval study requirements [28]. In 2018, Health Canada and Canadian Agency for Drugs and Technologies in Health announced the intention to co-develop an action plan to improve the process for using and integrating real-world evidence into regulatory and reimbursement decision-making [76]. Overall, there is variability in policies concerning the use of real-world evidence across different national agencies [77].

## 5. Considerations for Harnessing Real-World Evidence for Cancer Medicines Research

To maximize the potential of real-world data in cancer medicine research, practical issues need to be considered, including heterogeneity and limitations of real-world data sources and optimizing the conduct of real-world evidence research to improve its reliability and acceptance.

### 5.1. Heterogeneity of Real-World Data

In countries with universal public health systems, the strength of routinely collected health administrative data lies in its comprehensive coverage of heterogenous, real-world populations. The main limitation of these data is the absence of relevant clinical details, such as patient and cancer characteristics, constraining the analyses that can be conducted. Health administrative data are collected for administrative or financial reasons, not clinical care or research purposes, so the advantages of a large sample size and population-wide coverage of this data source are offset by the unavailability of variables that are not directly related to medicine dispensing and health service provision.

EHR are a source of highly granular clinical data but may not be collected in a structured format that is easily extracted and validated for secondary research. Different EHR programs are often used across different jurisdictions and parts of the health system, with little interoperability limiting the ability to provide comprehensive population-wide, EHR-derived clinical data. Disease-specific registries and databases are often established for research and/or epidemiological purposes and may include many clinically relevant variables that have been defined with research end points in mind but may not necessarily provide population-level coverage.

In short, there is no perfect, comprehensive real-world data source that includes all clinical variables for patients at a population-wide level, and there are often trade-offs between having a large, representative sample and the availability and completeness of research-relevant data points. Each data source has strengths and limitations that need to be considered when designing and interpreting evidence derived from real-world data.

### 5.2. Promoting the Quality and Reliability of Real-World Evidence

Standards for research using real-world data are not as mature as those for RCTs, leading to doubts about the reliability of studies using real-world data. Table 3 summarizes concerns about the quality and acceptability of real-world research, as well as potential solutions to address these concerns.

### 5.3. Concerns about the Reliability of Real-World Evidence

Uncertainty regarding the internal validity of studies, datasets of uncertain quality and opaque reporting of conduct and results may contribute to the lack of confidence from stakeholders and decision-makers regarding the reliability of studies using real-world data [78]. The increasing availability and use of big data for observational research also raises the risk of multiple hypotheses testing and fishing for positive associations [79]. Large volumes of data do not address design bias and defects and could actually exacerbate these issues if high precision is reported around the wrong answers [80]. For clinicians, policymakers and other research end users that are more familiar with the statistical rigour and transparency of a well-designed and well-conducted RCT, there may be concerns about the credibility of real-world evidence [56]. Patients may have misgivings relating to consent and privacy regarding the use of routinely collected health data for secondary research, signalling a need for greater clarity around the protection of privacy and data ownership to promote trust and understanding about real-world evidence [81].

#### 5.3.1. Strategies to Promote the Reliability and Credibility of Real-World Evidence

Given the uncertainties regarding the reliability of real-world evidence, there needs to be a better understanding of what high-quality, real-world evidence research looks like and the promotion of strategies for improving its quality and credibility (Table 3). Firstly, clinical questions should be meaningful, well-defined and answerable with available real-world data, rather than scenarios for which RCTs are necessary and feasible, such as when establishing the initial efficacy of novel therapies. Secondly, robust real-world evidence relies on real-world data that are high-quality, relevant and fit-for-purpose [82]. High-quality real-world data should be representative of the population of interest and contemporary clinical practice and have clear documentation of data completeness and the provenance of each data point [83]. Thirdly, study designs should be appropriate for answering the clinical question, take into account any data limitations, and avoid common design flaws by addressing immortal time bias, balancing measured and unmeasured confounders and including sensitivity analyses [84]. To ensure the internal validity of real-world studies, researchers should be mindful of controlling for potential sources of bias in real-world studies that may arise from provider-patient dynamics, data collection and processing techniques and differences in practice due to regional variations in standards of care and access to care [85]. Finally, to increase the confidence in the results of real-world evidence studies, study methodology needs to be clear, transparent and replicable.

Pre-registration of protocols is a standard requirement for conducting RCTs, and this approach is increasingly being adopted for real-world data research to reduce the risk of multiple hypotheses testing within studies, improve methodological transparency and support replication efforts [69,79]. Pre-registration can also help to address the community-level multiplicity issues that arise when multiple hypotheses are cumulatively tested by different researchers within a real-world database, by minimizing the effects of selective reporting and allowing researchers to identify all previously tested hypotheses [86]. ISPOR-ISPE have recently launched a Real-World Evidence Registry to facilitate pre-registration of real-world research protocols and promote trust in study results [87]. Elements of ideal protocols reflect a robust real-world research design: pre-specified hypotheses, analysis plans that are appropriate for the research question and account for biases and missing data, adequate capture of required data elements, clear documentation of data handling procedures, and transparent and traceable processes that allow replication and auditing [82].

#### 5.3.2. Guidance for Conducting Real-World Evidence Studies

Guidelines for real-world evidence development have been published to promote good practices and assure the public of the integrity of the research process and enhance confidence in evidence derived from these studies. The ISPOR-ISPE guidelines make recommendations on good procedural practices for real-world data studies of treatment and/or comparative effectiveness [78]. STaRT-RWE is a structured template for planning and reporting real-world evidence studies of the safety and effectiveness of treatments to guide the design and conduct of reproducible real-world evidence studies [88]. The RECORD statement relates to reporting of studies conducted using observational routinely-collected health data [89]. Frameworks and guidelines developed by regulatory bodies provide further guidance to the optimal conduct of real-world evidence studies, especially with regards to evidence generation for cancer therapies and pharmaco-epidemiologic safety studies [28,76,90,91,92,93].

## 6. The Potential of Real-World Data to Advance Cancer Medicine Research around the World

The potential of real-world data to facilitate oncology research relies partly on the health system organization. For instance, Australian health data holds considerable potential for real-world cancer research by virtue of the Australian health system. Healthcare in Australia is delivered through a comprehensive, public-funded, universal healthcare system, which includes the Commonwealth funding of cancer medicines that are recommended for public subsidy via the Pharmaceutical Benefits Scheme (PBS) following a cost-effectiveness assessment [94]. Routinely-collected health administrative dispensing data about publicly-subsidised cancer therapy could facilitate population-wide cancer medicine research at a national level. This contrasts with real-world data sources from other countries that do not offer national coverage, such as data collected by the provincial health systems in Canada [95], or data collected in the USA about specific subgroups based on their age or insurance status including the SEER-Medicare program [96], or commercial databases comprising private health insurance program enrollees [97,98]. In countries with data collections that cover population subsets, patient information may be incomplete as they move between health care providers for geographic, financial or demographic reasons, whereas the strength of Australia’s national health system lies in its capacity to collect comprehensive and longitudinal cancer medicine data for all residents. Canada has recently tried to capitalize on its robust province-based real-world data research track record by launching the Canadian Data Platform under the Strategy for Patient-Oriented Research (SPOR) to facilitate multi-jurisdictional research using various federal and provincial data sources [99].

Intravenous cancer medicines, which make up the majority of systemic cancer therapies, are typically administered in infusion clinics in outpatient settings attached to hospitals. In Australia, the PBS funds intravenous treatments that are delivered through hospital outpatient services, as well as oral cancer medicines that are dispensed through community pharmacies, thereby enabling the comprehensive capture of systemic cancer treatments. In other countries with comparable health systems, such as Canada and the Nordic countries, intravenous cancer treatments delivered through hospitals are not routinely captured by national medicine prescribing or dispensing data collections [100,101]. Some countries have recently introduced initiatives to facilitate a population-wide capture of systemic cancer therapy use, such as the Systemic Anti-Cancer Therapy database in the United Kingdom, which mandates submission of systemic cancer treatment data from hospital electronic prescribing systems from all National Health Service Trusts from 2014 [102]. In Norway, the INSPIRE project was established to include cancer medicine data from hospital systems as part of the Cancer Registry [103]. However, these new data collections involve dedicated data extraction processes and are contingent on the quality and completeness of hospital-based electronic prescribing systems, as opposed to the routine nature of PBS data collection in Australia. Table 4 describes ideal features of real-world data to enable cancer medicine research and compares the degree to which data sources from different countries align with these characteristics.

## 7. Conclusions

Although RCTs are the gold standard for generating evidence on cancer therapies with robust internal validity, there remain evidence gaps relating to patients, clinical practice and outcomes. Real-world data plays an important role in generating evidence that is complementary to conventional RCT evidence and improving clinical trial design. It is vital to understand the strengths and limitations of real-world evidence and RCT evidence to implement a framework in which they are used complementarily to create a robust evidence base for improving cancer care and guiding population-level decision making.

## Figures and Tables

**Table 1:** Strengths and limitations of clinical trials and studies leveraging real-world data.

	Strengths	Limitations
Clinical trials	Gold standard for assessing efficacy Provide precise measures of treatment efficacy and acute toxicity of new treatments under ideal conditions Process of randomization in randomized trials promotes even distribution of confounders between treatment groups Conducted by well-established methodological rules High internal validity, i.e., provides robust comparison between intervention and control	Costly, cumbersome and resource-intensive Limited number of patients Low external validity due to: - Highly selected participants due to strict inclusion and exclusion criteria - Under-representation of groups such as older adults and people with comorbidities - Protocols that may not reflect typical care - Use of surrogate endpoints that may not be valid measures of patient benefit Limited ability to detect rare and long-term side-effects
Real-world data studies	High external validity due to inclusion of large numbers of patients in routine care Provides evidence of effectiveness of new treatments in the general population, including those under-represented in clinical trials Can be performed relatively quickly and at low cost once data infrastructure is established Large sample sizes allow identification of rare events Facilitates long follow-up of patients	Susceptible to confounding and selection bias as interventions are not randomised Methodologically difficult to do well Variable data quality - Data may not include key clinical details, e.g., treatment plans, comorbidities, performance status - Higher levels of missing data

**Table 2:** Types of real-world data and their strengths, limitations and potential applications.

Type of Real-World Data	Types of Outcomes and Measures	Strengths	Limitations	Potential Applications
Electronic medical records	Patient-level clinical information, e.g., vital signs, primary diagnosis, medical history Medicine prescriptions Laboratory and imaging test results	Detailed patient-level clinical information Longitudinal data capture	Unstructured data is not easy to query and capture for research Does not include data on whether treatment orders (e.g., medicine prescriptions) were carried out	Understanding patient and disease characteristics, treatment and outcomes in routine clinical practice
Health administrative data	Medicine dispensing records Health service utilization (e.g., diagnostic tests, imaging, physician visits, hospital admission, emergency department use) Financial data	Large numbers of patients Near-complete history of a patient’s health care resource utilization and costs provided by a given payer (e.g., universal health system) Longitudinal data capture Representative of the population	Lack of clinical data Important clinical endpoints (e.g., progression, death) often unavailable Data quality issues	Measurement of health care utilization and costs Identify outcomes of patients with rare events
Cancer registries	Date of cancer diagnosis Location, histology and staging of cancer Demographic details of patients	Specific and uniform capture of clinically rich and defined patient characteristics and outcomes	Data is limited to the specific disease that the registry is designed to capture	Understanding the natural history of a disease Studying the changes in incidence and prevalence of conditions over time
Health surveys	Self-reported health status Health-related behaviours	Representative of the general population, if correctly weighted	Self-reported data may be affected by recall bias	Understanding the health and sociodemographic characteristics of a population
Patient-generated data	Health statistics (e.g., heart rate, step count, activity levels) Patient-reported outcomes Quality of life data	Capture detailed and longitudinal information including patient-reported outcomes Data contains patient-centric outcomes Does not rely on data reported by providers	Populations may not be representative Capture of limited outcomes	Direct measurement of patient symptoms, activity and other outcomes
Social media	Patient-reported outcomes and experiences	Unfiltered reporting of outcomes and experiences by patients	Usually limited to qualitative data Data not uniformly reported by patients Difficult to confirm clinical outcomes and authenticity	Understanding patient experiences (e.g., factors affecting treatment adherence)

**Table 3:** Concerns regarding studies using real-world data and strategies to promote the quality and acceptability of real-world evidence research.

**Concerns regarding real-world evidence research** Limited availability of relevant research end points because data is not collected for primary research purposes Higher rates of inaccurate and incomplete data compared to RCTs Unmeasured confounders leading to residual confounding in studies of treatment effectiveness Unclear and difficult to replicate study methodology Spurious results arising from multiple hypothesis testing and fishing for positive associations Insufficient safeguards for protecting patient privacy and data ownership
**Strategies for improving the quality and acceptability of real-world evidence research** Selection of meaningful, well-defined clinical questions that are appropriate for investigating using real-world data Promote use of real-world data that are high-quality, relevant and fit-for-purpose Clear documentation of data provenance and completeness Appropriate study design that takes into account data limitations and controls for potential sources of bias Pre-registration of protocols with pre-specified hypotheses and analysis plans that allow for auditing and replication Clear legislation/provisions around data ownership and protection of privacy Dissemination and adoption of guidelines and frameworks promoting good research practice for real-world evidence studies Guidelines for clear and comprehensive reporting of real-world evidence studies

**Table 4:** Comparison of health administrative data collections from selected countries and alignment with ideal characteristics of real-world data for cancer medicine research.

Country	Universal Health Care System	Nationwide Coverage	Routine Collection of Cancer Medicine Data	Datasets for Facilitating Real-World Cancer Medicine Research
Australia	Yes	Yes—Commonwealth health system components (PBS, MBS)	Yes—all PBS-subsidised cancer medicines given in outpatient settings	Yes—DVA data collection
Canada	Yes	No—data collected in province-based health systems	Yes	Yes—SPOR to coordinate cross-jurisdictional research (but not cancer specific)
UK	Yes	Yes	No	Yes—SACT database collecting mandatory reporting of cancer treatment from NHS hospitals
Nordic countries	Yes	Yes	No	Yes—INSPIRE to collect cancer medicine data as part of Cancer Registry in Norway
USA (SEER-Medicare)	No	No—SEER registries cover 47.9% of the USA population [104], 96% of SEER patients aged ≥ 65 years can be matched to Medicare data [105]	Yes—Medicare data includes claims for episodes of systemic cancer treatment.	Yes—SEER-Medicare, CancerLinQ

CancerLinQ: Cancer Learning Intelligence Network for Quality, DVA: Department of Veterans’ Affairs, INSPIRE: INcreaSe PharmaceutIcal Reporting, MBS: Medicare Benefits Schedule, NHS: National Health Service, PBS: Pharmaceutical Benefits Scheme, SACT: Systemic Anti-Cancer Therapy, SEER: Surveillance, Epidemiology and End Results, SPOR: Strategy for Patient-Oriented Research.
